# Refractory Metal Coated Alumina Foams as Support Material for Stem Cell and Fibroblasts Cultivation

**DOI:** 10.3390/ma14112813

**Published:** 2021-05-25

**Authors:** Georg Hasemann, Ulf Betke, Manja Krüger, Heike Walles, Michael Scheffler

**Affiliations:** 1 Institute of Materials and Joining Technology, Otto-von-Guericke-University Magdeburg, Universitätsplatz 2, 39106 Magdeburg, Germany; ulf.betke@ovgu.de (U.B.); manja.krueger@ovgu.de (M.K.); m.scheffler@ovgu.de (M.S.); 2 Institute of Chemistry—Core Facility Tissue Engineering, Otto-von-Guericke-University Magdeburg, Pfaelzerstr. 2, 39106 Magdeburg, Germany; heike.walles@ovgu.de

**Keywords:** ceramic foams, metal coating, biomaterials, tissue engineering, stem cell cultivation

## Abstract

Ceramics are widely used as implant materials; however, they are brittle and may emit particles when used in these applications. To overcome this disadvantage, alumina foams, which represent a 3D cellular structure comparable to that of human trabecular bone structures, were sputter coated with platinum, tantalum or titanium and modified with fibronectin or collagen type I, components of the extracellular matrix (ECM). To proof the cell material interaction, the unmodified and modified materials were cultured with (a) mesenchymal stem cells being a perfect indicator for biocompatibility and releasing important cytokines of the stem cell niche and (b) with fibroblasts characterized as mediators of inflammation and therefore an important cellular component of the foreign body reaction and inflammation after implantation. To optimize and compare the influence of metal surfaces on cellular behavior, planar glass substrates have been used. Identified biocompatible metal surface of platinum, titanium and tantalum were sputtered on ceramic foams modified with the above-mentioned ECM components to investigate cellular behavior in a 3D environment. The cellular alumina support was characterized with respect to its cellular/porous structure and niche accessibility and coating thickness of the refractory metals; the average cell size was 2.3 mm, the average size of the cell windows was 1.8 mm, and the total foam porosity was 91.4%. The Pt, Ti and Ta coatings were completely dense covering the entire alumina foam surface. The metals titanium and tantalum were colonized very well by the stem cells without a coating of ECM components, whereas the fibroblasts preferred components of the ECM on the alumina foam surface.

## 1. Introduction

Artificial prosthesis have been successfully implanted in the human body for many years and improve mobility, vitality and the quality of life of many patients. A large variety of different metallic and ceramic implant materials are in clinical use. However, there is ongoing demand and scientific interest to improve performance and longevity of implants in terms of tribological performance, wear resistance, corrosion and bio- and tissue compatibility to prevent, i.e., inflammation reactions. Thus, a variety of materials and composites are of interest for certain implant applications.

Materials available for total hip arthroplasty (THA) are for examples metal-on-highly-cross-linked-polyethylene (MoP), ceramic-on-highly-cross-linked-polyethylene (CoP), metal-on-metal (MoM) and ceramic-on-ceramic (CoC) bearing couples. In general, they show good bio- and cell compatibility. A drawback is, however, the wear resistance causing surface erosion and erosion particles that can lead to implant loosening. Inflammatory cell response and fibrotic tissue reactions are symptomatic around CoCs, specifically if alumina-toughened zirconia (ATZ) is used as implant material [1]. As a result, additional surgical efforts are often necessary to fix the issue [2] and may involve revision surgeries with high complication rates and high technical demands [3], they are expensive [4] and have usually a low level of satisfaction for the patients [5]. Besides, Ti-Al-V [6,7], Ti-Mo-Zr-Al [8], Co-Cr-Mo [9,10] and porous Ta [11,12] implants possess also very good bio- and cell compatibility [6] and are used in a large variety of implant applications, i.e., as hip, shoulder or dental implants. But even these implant “workhorses” may promote inflammation and fibrous tissue reactions due to metal ion release. Cytotoxic metal ions can cause damage to osteoblasts that can no longer form bone material. Thus, a hindered local ingrowth impairs osseointegration of implants and are a major aspect for aseptic loosening [13]. In future, special implant treatment such as Plasma Immersion Ion Implantation (PIII) may help to avoid fibrosis and a heavy foreign body reaction of the immune system on implants and implant materials [14].

In this study a functionalized cellular ceramic-metal material is provided which might be suitable as replacement for the trabecular bone and may have synergetic effects with state-of-the-art implant materials. The present study aims to gain inspiration from other implant groups, i.e., for hips, knees or dental implants, to cross-link both developments and demands for new and innovative biomedical materials solutions. The materials design strategy is based on classic implant materials and geometries. The ceramic-metal functional material will be designed as a macroporous, cellular material which is beneficial in terms of implant ingrowth [15]. Cellular alumina supports have been used that almost perfectly represent trabecular bone-like artificial structures. However, due to their brittleness, ceramic cellular scaffolds may emit particles when used in biomedical applications [16]. For better cell and tissue compatibility, the macro-porous alumina supports were metallized with thin layers of bioactive materials such as Pt, Ti and Ta. First cell population experiments on metallized alumina supports were performed with mesenchymal stem cells (MSCs) since they represent the most relevant cell type in the bone marrow niche and have the potential to regenerate multiple cell types. The bone marrow contains additionally non-haematopoietic, so-called stromal cells, like osteoblasts endothelial cells, or fibroblasts [17]. Fibroblasts can induce inflammation by the secretion of chemokines in response to the implanted material [18], and it is underlined by multiple publications that fibroblast are important cellular components in the pathogenesis of diseases, such as cancer [19].

To proof vitality population capacity of fibroblasts and hematopoietic stem cells (HSCs), these cell types were additionally cultured on the new metallized alumina scaffolds. The fibroblasts (FBs) are an important indicator for the foreign body reaction and inflammation after implantation. The HSCs can be used in future to establish co-cultures of both stem cell populations of the bone marrow niche, since MSCs are a rather robust cell type and the HSCs are more sensitive, and thus a more critical parameter to test the bioactivity and cell ingrowth behavior of this new material.

## 2. Materials and Methods

### 2.1. Preparation of Cellular Alumina Supports

The alumina foams used as support for the stem cell cultivation were prepared by the sponge replication, or Schwartzwalder technique [20]. A polyurethane foam template was coated with an alumina dispersion; the dried green body was subsequently heat-treated for removal of the template and sintered to consolidate the ceramic structure.

For the preparation of the alumina dispersion 100.0 g Al_2_O_3_ powder (CT 3000 SG, d_50_ = 0.5 µm; Almatis GmbH, Ludwigshafen, Germany), 25.0 g demineralized water and 1.0 g ethylammonium citrate-based deflocculant (Dolapix CE64; Zschimmer & Schwarz Chemie GmbH, Lahnstein, Germany) were homogenized in a planetary centrifugal mixer operated at 2000 rpm for 15 min (THINKY Mixer ARE-250; THINKY Corp., Tokyo, Japan). Subsequently, 1.5 g polyvinylalcohol-based binder (Optapix PA 4G; Zschimmer & Schwarz GmbH, Lahnstein, Germany) and 0.1 g anti-foaming agent (Kontraspum K 1012; Zschimmer & Schwarz GmbH, Lahnstein, Germany) were added to the dispersion and a second homogenization cycle was performed. After this, the resulting alumina dispersion possessed a solid loading of 78.3 wt.%, which is 47.8 vol.%, and was suitable for application onto the polyurethane templates.

Reticulated polyurethane (PU) foams with a cell count of 20 ppi (pores per linear inch; SP30P20R; Koepp Schaum GmbH, Oestrich-Winkel, Germany) in the dimension of 15 mm × 15 mm × 20 mm were used as template structures. Each PU foam was completely loaded with the alumina dispersion and freed from the excess amount by squeezing it carefully until the coated template had a weight of 1.5 ± 0.05 g. After drying at room temperature, the foams were transferred into a circulating air furnace (KU 40/04/A; THERMCONCEPT Dr. Fischer GmbH, Bremen, Germany) and the PU templates were thermally removed (110 °C/2 h, 250 °C/3 h, 400 C/h, heating/cooling rate 1 K∙min^−1^). Subsequently, the samples were transferred into a sintering furnace (HTL 10/17, THERMCONCEPT Dr. Fischer GmbH, Bremen, Germany) and were densified at 1650 °C for 3 h.

### 2.2. Deposition of Metallic Thin Films on Glass Coverslips and on Cellular Alumina Supports

The metallization with platinum, titanium and tantalum was performed by Ar plasma-assisted sputter coating. In a first approach, five glass coverslips were loaded into the sputter coater (Q150T ES; Quorum Technologies Ltd., Laughton, UK), which was equipped with a sputter target made of pure platinum, titanium or tantalum, respectively. The metallization was performed for three minutes and a plasma current of 100 mA. Subsequently, all foams were turned upside down and the coating procedure was repeated using the conditions as adjusted above. Secondly, five Al_2_O_3_ foams were loaded into the sputter coater and metallized using the same parameters as used for the coverslips.

Thus, the experimental procedure allows to study the vitality of the stem cells on plain 2D metalized surfaces compared with the 3D environment of porous alumina foams, which represents the technical (artificial) stem cell niche.

### 2.3. Characterization of the (Metallized) Cellular Alumina Supports

The total porosity of the as-prepared supports (V_pores_/V_foam_) was calculated from the geometric foam density (foam mass m_f_ divided by the geometric foam volume V_f_) in relation to the theoretical density of the strut material (3.94 g·cm^−3^ for alumina; ref. [21]). The results of ten samples were averaged. The surface morphology of the pristine, as well as Pt-, Ti-, and Ta-coated foams was investigated using scanning electron microscopy (SEM); a XL30 ESEM-FEG microscope (FEI/Philips, Hillsboro, OR, USA) equipped with a secondary electron (SE) and backscattered electron (BSE) detector. The elemental composition of the strut material was analyzed by energy-dispersive X-ray spectroscopy (EDS, EDAX-AMETEK GmbH, Weiterstadt, Germany). Estimation of the Pt-, Ti-, and Ta-coating thickness was performed on selected, epoxy resin-embedded samples which were beforehand grinded and polished with 3 µm and 1 µm diamond dispersions, respectively.

Exemplary for the metallized sputter coatings, the tantalum layer thickness was estimated via EDS measurements using different acceleration currents for the primary electron beam. From the obtained EDS spectra, the Ta concentration was determined by using the software EDAX-Genesis. In addition, the information depth of the EDS measurement *R*_E_ was calculated according to the Thomson–Whiddington relation (Equation (1)):

(1) $$R_{E}=\frac{U^{2}}{\rho b}$$

In Equation (1) *U* is the acceleration voltage for the primary electron beam, *ρ* is the material density (a density of 4.0 g·cm^−3^ has been assumed for all calculations) and the constant *b* is 4 × 10^11^ cm²·eV²/g. The Ta concentration obtained from the EDS spectra is then plotted as a function of the estimated information depth. The data are fitted by a logistics function c_Ta_ = a·*R_E_*^b^ whereas a is 23 ± 1.1 µm^−2^ and the exponent *b* is −0.56 ± 0.03.

For the cell size determination of the foams a microcomputer tomograph (µ-CT, nanotom S, GE Sensing & Inspection Technologies, Wunstorf, Germany) was used for data aquisition and the software CTAnalyser V.1.18 (Bruker microCT, Kontich, Belgium) was used for analysis of the tomographic data. The voxel size was adjusted to a resolution of (10 µm)^3^. From the structure separation distribution histograms obtained from CTAnalyser, the cell size of the respective sample was extracted by applying a fit with a set of Gaussian functions. For details of the tomographic characterization and the morphology analysis using CTAnalyser [22,23].

The surface of the original as well as the metallized alumina foams was further investigated by Raman spectroscopy using a WITec Alpha 300R Raman microscope (Ulm, Germany) equipped with a 532 nm laser. Spot measurements were performed on selected samples and ten Raman spectra were collected per spot and finally averaged.

### 2.4. Cell-Biological Characterization of Cultivated (Metallized) Cellular Alumina Supports

Sterile work was conducted in a class II laminar flow bench. If necessary, all solutions were sterile filtered before use. All materials, except single-use items, were autoclaved or hot-air sterilized before use. If not otherwise stated, all cell culture media and solutions were warmed up to 37 °C in a water bath. Culture of cells was performed in incubators with a relative humidity of 95%, 5% CO_2_ and a temperature of 37 °C.

Fibroblasts were isolated from fascia biopsies of donors with different ages. A human stem cell bank of hematopoietic stem cells (HSCs) and bone marrow mesenchymal stromal cell (MSC) was established at the University of Wuerzburg [24]. All primary cell isolation and cultures were performed under the approval of the Local Ethics Committee of the University of Wuerzburg (182/10) and informed consent of the patients. HSCs were expanded in StemPro-34 (10639011, Gibco, Waltham, MA, USA), Fibroblasts in Dulbecos Medium (DMEM 61965059, Gibco) supplemented with 10% *v*/*v* fetal calf serum (FCS 10082147, Gibco), and MSCs in mesenchymal stem cell medium kit (MSCGM-CD PT-3001 Lonza) supplemented with 2% *v*/*v* FCS (10082147, Gibco) and 1% *v*/*v* Penicillin/Streptomycin (10378016, Gibco). MSCs of all donors were characterized for the expression of surface markers CD29, CD44, CD73 (Sh3/4), CD90 Thy1 through flow cytometry. All donor cells lacked the expression of the CD14 and CD45. Once 80% confluence were reached, all cell cultures were passaged by a treatment with 0.05% Trypsin/EDTA solution (15400-054, Invitrogen) at 37 °C for 3–5 min. The detachment of the cells was confirmed by microscopy and the trypsin reaction was stopped by adding FCS. The cell suspension was centrifuged in a 50 mL centrifuge tube at 1200 rpm for 5 min. After resuspending the cells in fresh medium and counting the cells, they were reseeded at their respective densities on the 15 × 15 mm^2^ glass-metal coverslips or ceramic foams.

**Figure 1:**
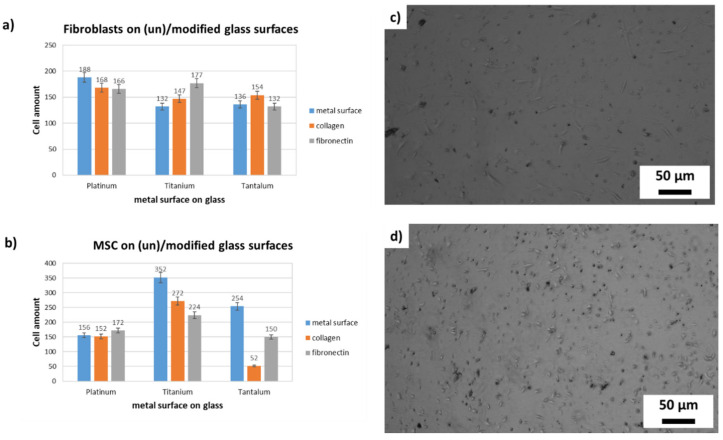
FBs and MSCs on unmodified or with ECM components modified metalized surfaces; To identify the optimal metal substrate, primary human Fibroblasts (**a**,**c**) and human mesenchymal stem cells (**b**,**d**) (MSC) were cultured for one week on platinum, titanium and tantalum surfaces. The cells grow either in direct contact to metallic surfaces or on collagen I or fibronectin coated coverslips.

To prove the influence of different metallized alumina scaffolds, 15 × 15 mm^2^ glass coverslips (272-KKA2.1, Carl Roth, Karlsruhe, Germany) sputter-coated with platinum, titanium and tantalum were sterilized and then either directly used for cell culture or modified with collagen I or fibronectin. To modify the metal surface with these extracellular matrix (ECM) components, they were incubated at 37 °C for 24 h in a 0.5 mg/mL ECM-protein solution. Thereafter they were transferred in a 12 well plate and immediately seeded with 1 × 10^4^ cells/well. All seeding experiments were replicated three times (*n* = 3) with cells of the same donor and passage. At the end of the experiments 10 pictures of each coverslip were taken, cells counted per picture (Figure 1c,d) and the total amount on the 15 × 15 mm^2^ area calculated (Table 1).

Cellular behavior on the 15 × 15 mm^2^ metal modified glass surfaces or the metal modified ceramic foams was monitored by modifying the cells with nontoxic fluorescent colors using CellTracker fluorescent probes (Green CMFDA (C7025) or RED CMTPX (C34552), Invitrogen, Carlsbad, CA, USA). Additionally, actin filaments of the cells were stained with CytoPainter Phalloidin-iFluor 647 reagent (ab176759, Abcam, Cambridge, UK) and the nuclei of the cells were blue counterstained with blue 4′,6-diamidino-2-phenylindole (DAPI) (D1306, ThermoFischer, Waltham, MA, USA). If necessary, cells were fixed on the surface by an incubation in 30 mL 4% formalin (256462, Applichem, Glenview, IL, USA) for 12 h at 4 °C.

To investigate cell viability in the 3D culture, a quantitative 3-(4,5-dimethylthiazol-2-yl)-2,5-diphenyltetrazolium bromide (MTT) (ab146345, Abcam) assay was performed. For this purpose, the medium was aspirated and replaced with 1 mg/mL MTT solution. After incubation at 37 °C for 3 h, the formazan was extracted with isopropanol. Absorption was measured at a wavelength of 570 nm and a reference wavelength of 630 nm.

## 3. Results and Discussion

Pitkänen et al. [25] worked out the contradicting results on the osteoinductive properties of β-TCP and were speculating that they might vary according to the surface properties of the material [26,27]. The brittle and hard calcium phosphates are very difficult to shape and implant. Synthetic polymers, i.e., polylactide, polyglycolide, poly-ε-caprolactone, on the other hand, support this osteogenic differentiation of stem cells leading Pitkänen et al. to a β-TCP/PLCL biocompatible composite.

The present study introduces another approach for biocompatible materials that is based on metallized alumina foams. These functionalized alumina scaffolds aim to investigate their influence on the long-term culture of stem cells and the potential for induction of inflammation and foreign body reactions.

To carry out this work, the cell-compatibility of Ti, Ta, and Pt metallized glass and alumina supports were tested. Planar 15 × 15 mm^2^ glass coverslips were used as model system for identifying the optimal metal substrate for the MSC and fibroblast cultivation. The cultivation experiments were performed with and without the selected ECM components collagen and fibronectin on 15 × 15 mm^2^ coverslips. It has been reported in literature that the ECM regulates cell differentiation, growth and migration [28,29] and that collagen type I is the most abundant protein of the ECM [30]. Fibronectin, however, is important for the cell-material interaction [28] and can induces the cellular deposition of collagen [29]. The best combinations of metallization and ECM were selected for further investigation of the cell proliferation on cellular alumina substrates.

### 3.1. Bio- and Cell Compatibility of Metalized Layers

In the next series of experiments, we evaluated the ideal surface for either fibroblasts, as a typical stromal cell type and mesenchymal stem cells, a representative of the natural stem cell niche in the human body and responsible for the long life regeneration of all cell types responsible for bone healing and re-modeling. Yuan et al. [31] showed that physicochemical and structural characteristics significantly affect the induction of cellular differentiation of MSC. Additionally, microporosity is a key feature to induce bone in-vivo formation [32].

To evaluate the ideal surface for cell proliferation 15 × 15 mm^2^ the glass coverslips were modified with platinum, titanium and tantalum and incubated with components of the extracellular matrix (ECM), collagen I or fibronectin, see Figure 1.

**Figure 2:**
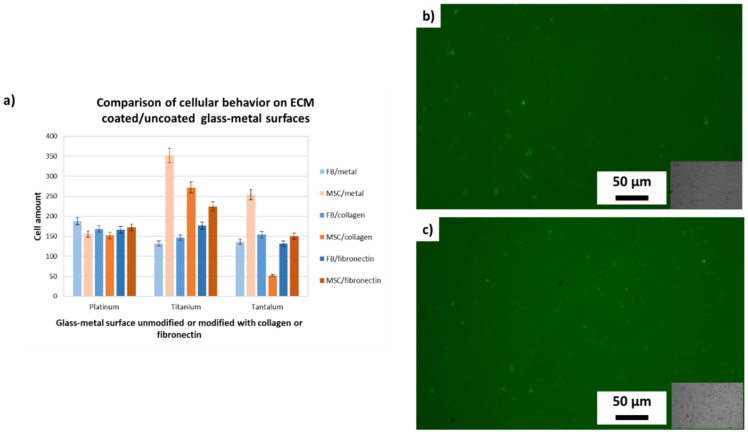
(**a**) Comparison of cellular behavior on different glass-metal surfaces cells (*n* = 3) as above, but fluorescent (GFP labeled), (**b**) FBs and (**c**) MSCs.

It is noticeable that the metals titanium and tantalum have been colonized very well by the stem cells without being coated with ECM components, whereas the fibroblasts preferred surface components of the ECM. The all over aim of this study is to develop ceramics with metal surfaces which can be populated by stem cells. As shown in Figure 2, MSCs prefer titanium or tantalum surfaces without any ECM components, whereas the FBs show better proliferation if the metal surface is coated with collagen I or fibronectin. All over, the FBs show the highest proliferation rate on the platinum surface, whereas the stem cells have a significantly higher proliferation on titanium or tantalum surfaces.

Based on these results, the surface of the porous ceramic supports were further modified with titanium and tantalum.

In addition, it was possible to show that, in all experiments, the fluorescence marking of the cells used in the 2D culture did not facilitate the determination of the number of cells over time. This is probably due to reflections on the metallic surfaces.

### 3.2. Characterization of Cellular Alumina Supports

Several groups reported that bone implants with a highly porous structure enhance immigration of (stem) cells [33] and the cellular induced deposition of minerals, a prerequisite of bone regeneration [25] and bone marrow niche homeostasis [17].

Since cellular ceramics are of interest for improving the ingrowth of implants into the bone shaft, due to their resemblance to the inner structure of the human trabecular bone material, the cell cultivation on metallized alumina foams has been investigated. The MTT assay using the glass coverslips has shown that the stem cell population behaves similarly on Ti and Ta metalized surfaces, and the cell growth on the Pt coated supports is worse. Thus, these two elements were chosen for further investigations on the cellular alumina supports. In addition, for technical biomedical applications, platinum might be too expensive and is therefore not further investigated in this study.

**Figure 3:**
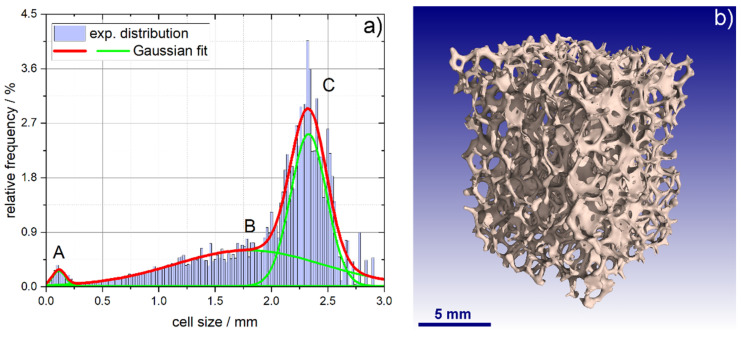
(**a**) structure separation distribution obtained from morphological analysis of µ-CT data on alumina foams; the maxima correspond to the hollow strut cavities (A), the cell windows (B; ~1.8 mm) and the actual foam cells (C; ~2.3 mm); (**b**) 3D rendering of the foam structure obtained from µ-CT data.

The total geometric porosity of the alumina foams is 91.5 ± 0.4% and the foam cell size amounts to 2.32 ± 0.16 mm according to a morphometric analysis of µ-CT data (maximum C in Figure 3a). The cell window diameter, which is an essential parameter with respect to the accessibility of the cell pore volume, is estimated to be 1.8 ± 0.6 mm (broad maximum B in Figure 3a). As an additional pore space, cavities inside the ceramic struts of the obtained foams are present, which result from the thermal removal of the PU foam template during manufacturing (diameter: 114 ± 48 µm; maximum A in Figure 3a).

### 3.3. Characterization of Metalized Cellular Alumina Supports

**Figure 4:**
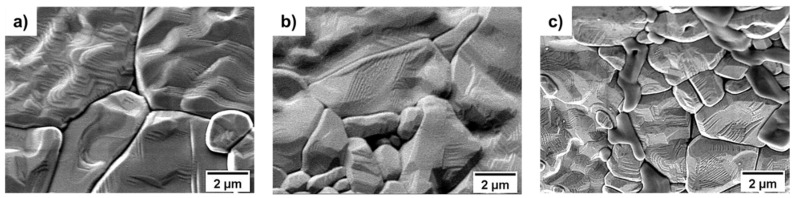
(**a**) SEM images of Al_2_O_3_ foam surfaces before sputtering, (**b**) Ta sputtered alumina surface and (**c**) Ta sputtered alumina surface after cell cultivation; the cells have been removed prior to SEM investigations.

After 3D characterization of the cellular Al_2_O_3_ supports, they were metallized via a sputter process. The same sputter parameters were used for both target materials. Figure 4 exemplary shows the Al_2_O_3_ surface before sputtering, sputtered with Ta and the Ta-sputtered surface after cell cultivation for 14 days.

**Figure 5:**
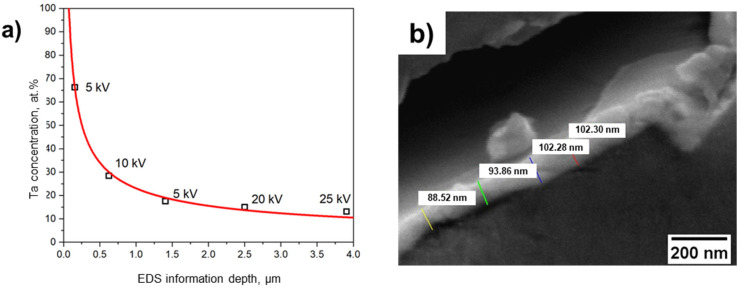
(**a**) Measured Ta concentration as a function of U to estimate the sputtered layer thickness on the Al_2_O_3_ foam surfaces, (**b**) measured Ta layer thickness at a metallographically prepared cross section.

Using the Thomson–Whiddington relation (Equation (1)), the Ta concentration obtained from the EDS spectra is then plotted as a function of the estimated information depth Figure 5a). The data are then fitted by a logistics function c_Ta_ = a·*R_E_*^b^ whereas a is 23 ± 1.1 µm^−2^ and the exponent b is −0.56 ± 0.03. The Ta coating thickness is then obtained by extrapolation of c_Ta_ to 100 at.% in form of the corresponding value for *R_E_* and could be determined to approximately 72 nm. However, this should be considered as a conservative estimation, as the calculation contains several assumptions and approximations. Nevertheless, this approach allows to estimate the layer thickness of samples where the cross section is not available. In Figure 5b) the layer thickness was directly measured using a metallographically prepared sample. Here, the sputtered Ta layer thickness was estimated to be around 100 ± 10 nm, respectively, and is in good agreement to the estimated value of 72 nm from the EDS data.

**Figure 6:**
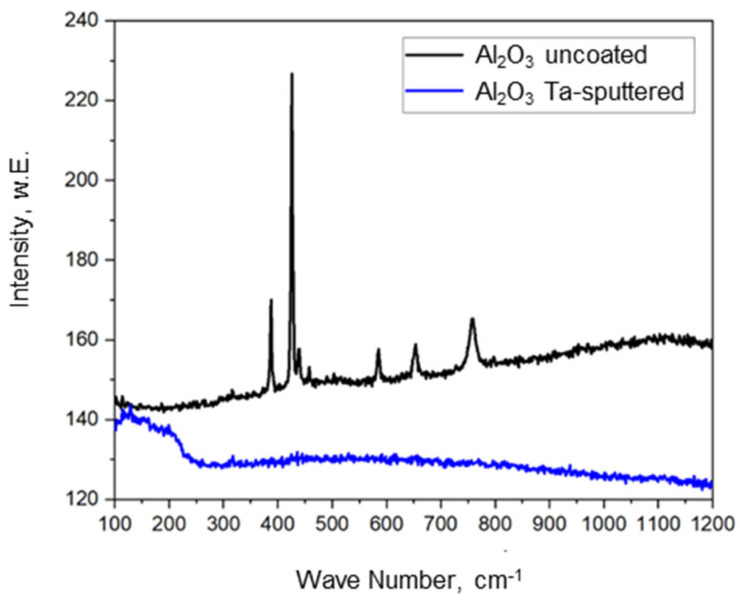
Raman spectra of alumina supports, non-metallized and metalized with tantalum.

The Ta layer deposition was further verified with Raman spectroscopy. Figure 6 shows the Raman spectrum with the typical vibrations between 350 and 800 cm^−1^ characteristic for the pure alumina support materials in direct comparison with the Ta-sputtered Al_2_O_3_ foam structure for which no Raman activity was detected. After metallizing with tantalum, the typical Raman bands for alumina disappear since pure metallic substances are usually non-Raman active. In addition, no signals corresponding to an oxidic tantalum species were detected. Thus, a pure metallic tantalum layer was deposited by sputter coating rather than a tantalum oxide species.

### 3.4. Cell Populations on Metalized Cellular Alumina Supports

**Figure 7:**
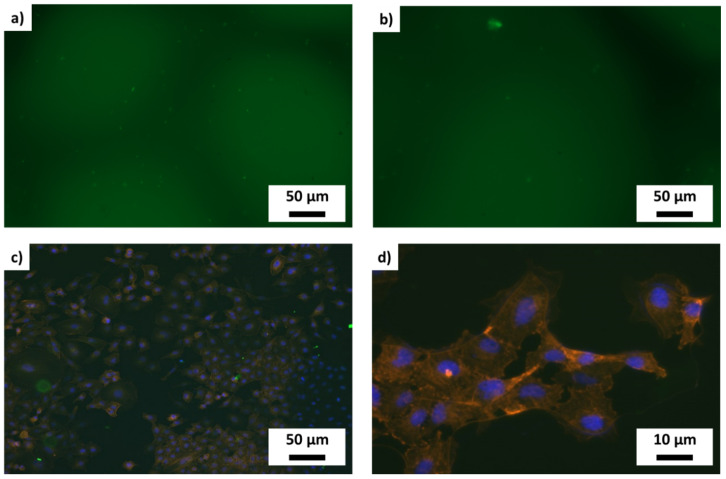
3D culture of stem cells on metal modified ceramic foams (functionalized) without coating of ECM components. Black shadows in (**a**) and (**b**) are the struts of the alumina foam scaffold. Green fluorescent cells on the black shadow are growing on the surface, whereas the lighter background shows cells grown in deeper areas in the foam. (**a**) on tantalum (**b**) on titanium—no discernible differences—(**c**): blue stained (Dapi) nuclei on formalin fixed samples. It is visible that there are many more cells on the surface than just appear in the green fluorescence—(**d**): enlargement of the formalin fixed cells on the surface—clearly to see are the blue nuclei and the red stained cytoskeleton.

Functionalized foams were populated with green fluorescent (GFP)-labeled MSCs with a cell density of 1 × 10^4^ cells/foam in 250 μL and cultured for 14 days. Every third day the medium was changed and a fluorescence image of the cells was taken. Due to the 3D structure of the intransparent alumina foam scaffolds, microscopy of the biological cells over time is a challenge, as shown in Figure 7a) for tantalum and Figure 7b) for titanium, each cultivated for one week. It is not possible to quantify the cell amounts at specific time points during the 3D culture. Thus, the cells were fixed at the end of the experiments, after 14 days in 3D culture. By means of the staining of the nucleus (Figure 7c,d) and the cytoskeleton (Figure 7d) it could be documented that the stem cells form a stable and confluent cell layer on the surface of the metallic modified ceramic foams. The staining of the cytoskeleton also proves that the cells are physiologically expanded and vital in the 3D culture. These results are a motivation for future work to establish methods for the semi-quantitative analysis of the amount of cells on the scaffold and their estimation of the immigration depth into the 3D scaffold.

In order to get the information on total cell amount of vital cells and immigration depth, despite of the aforementioned challenges, an MTT assay-based readout was conducted. After trypsination of the confluent MSCs, the cell density was adjusted to 5 × 10^3^, 1 × 10^4^, 5 × 10^4^, 1 × 10^5^, 5 × 10^5^, 1 × 10^6^ and 5 × 10^6^ on the sputtered 15 × 15 mm^2^ coverslips. The cell suspensions were pipetted into a 12 well plate, with the glass-metal plate on the bottom and cultured for 24 h at 37 °C. After the culture, an MTT assay (2D) was performed and the measured absorbance at 595 nm was used to create a standard curve.

In parallel, the titanium or tantalum modified or unmodified ceramic foams were populated with 1 × 10^4^ cell in 250 µL medium und cultured for 14 days. Every second day the medium was exchanged. On day 14 the 3D MTT assay was performed. Table 1 shows the absorbance at 595 nm for the MTT assay investigations on planar (glass (2D)) and 3D (metallized alumina scaffold) supports.

Using the MTT assay, it was possible to prove that the MSCs on titanium and tantalum modified ceramics show a very comparable growth behavior over two weeks, as summarized in Table 1. The MSCs grow almost equally well on titanium and tantalum modified alumina foams and about 20% less on the uncoated alumina foams. In contrast to the experiments on the metal-modified glass plates, there was no difference between the two medically interesting metal coatings tantalum and titanium. The number of cells in both 3D scaffolds was approx. 3 × 10^5^ cells, which corresponds to a surface of 20 mm^2^ populated with a confluent cell monolayer [20]. If one calculates this area with the size of the scaffolds (having and edge length of 15 mm) and the porosity of the sponges, it could be determined that the MSC grow approx. 0.25–0.44 mm into the scaffold.

The innovative combination of experimental scaffolds colonized with stem cells represent a promising strategy for regenerative healing of periapical and alveolar bone. Porous structures are seen as a key feature for the proper colonization of the scaffold by the cells. According to Tatullo et al. [33], the mineral compounds used to build the scaffolds should be homogeneously distributed. This aspect is important to have a homogeneous bioactivation of the cells along the whole structure of the scaffold. By using ceramic alumina foams as scaffold materials, this aspect is fully covered and the presented results can be taken as an experimental evidence.

In addition, the differentiation of stem cells, i.e., pulp stem cells (DPSCs), might be further influenced by different types of mechanical and hydrostactic loadings, as well as the surface topography [34]. This urges the need for further investigation on 3D scaffolds for stem cell cultivation to provide a habitable environment for different types of stem cells for various biomedical and implant applications.

## 4. Conclusions

Open cellular alumina foams were sputter coated with titanium, platinum and tantalum to obtain dense, approximately 100 nm thick metallic layers, and they were cultivated with mesenchymal stem cells or fibroblasts; the alumina foams were modified with ECM components prior to the cultivation and non-coated alumina foams, as well as metal-coated glass coverslips were cultivated for comparison and for semiquantitative cell density measurements. It was found that:

- Fibroblasts have a somewhat lower proliferation rate on the metal-coated and unmodified planar substrates, while the proliferation rate of the stem cells is somewhat lower at the ECM modified planar substrate, both over time. The number of cells per area were twice as high for stem cells compared to fibroblasts cultivated, both on titanium metal-coated, unmodified substrates.
- The cultivation process on planar substrates was transferred to open cellular metallized (titanium; tantalum) alumina foams and a semiquantitative analysis of the cell numbers on this non-planar scaffold was possible using an MTT assay with varying cell concentrations.
- This results in an almost equally proliferation rate of MSCs and FBs on the metallized alumina foams and an approximately 20% lower proliferation rate on the surface of the uncoated alumina foams.
- With these experiments it could clearly be shown that both, titanium and tantalum, are metals that are suitable for coating cellular ceramic materials for subsequent cell cultivation.
- Those coatings may reduce the release of particles from ceramic implants during integration into the human body.

## Figures and Tables

**Table 1:** Absorption at 595 nm of the MTT assay to calculate cell amount in 3D scaffolds.

Cell Amount (MSCs)	MSCs (2D) on Titanium Cover Slips (*n* = 3)	MSCs (2D) on Tantalum Cover Slips (*n* = 3)	3D Culture on Titanium (Ti) and Tantalum (Ta) Coated Ceramic (*n* = 3) Absorption/Cell Amount	3D Culture on Titanium (Ti) and Tantalum (Ta) Coated Ceramic (*n* = 3)—Estimated Covered Surface
0	0.203	0.203	-	-
5 × 10^3^	0.283	0.295	-	-
1 × 10^4^	0.414	0.407	-	-
5 × 10^4^	0.541	0.545	-	-
1 × 10^5^	0.888	0.858	0.984 (Ti)—3 × 10^5^ 0.971 (Ta)—3 × 10^5^	18.819 mm² 18.570 mm²
5 × 10^5^	1.059	1.092	-	-
1 × 10^6^	1.305	1.211	-	-
5 × 10^6^	1.437	1.252	-	-

## Data Availability

Data are part of ongoing studies and can not be shared at the present state.
